# Identification of an Unmet Medical Need: Height of Depression, Hypersomnia, and Sleep Apnea Positively Correlate With the Level of Fatigue in Patients With Immune Thrombocytopenia

**DOI:** 10.7759/cureus.47003

**Published:** 2023-10-13

**Authors:** Rosa S Alesci, Carola Hecking, Maike V Weissmann

**Affiliations:** 1 Blood Coagulation Centre, Medizinisches Versorgungszentrum (MVZ) Institut für Medizinische Diagnostik (IMD) GmbH, Bad Homburg, DEU; 2 Blood Coagulation Centre, Medizinisches Versorgungszentrum (MVZ) Institut für Medizinische Diagnostik (IMD) GmbH, Mannheim, DEU

**Keywords:** bdi questionnaire, facit f questionnaire, hypersomnia, obstructive sleeping apnea syndrome, quality of life, comorbidity, rare disease, depression, fatigue, immune thrombocytopenia

## Abstract

Introduction: Immune thrombocytopenia (ITP) is a rare chronic disease, frequently accompanied by fatigue, which is an important comorbidity associated with this disease. Patients experience difficulties in managing their daily activities and a reduction in their overall quality of life (QoL). The causes of fatigue in ITP are not clarified yet, and underlying causes seem to be multifactorial. The development of fatigue may not solely be influenced by a decrease in platelet count but also by unknown factors as well as psychological reasons.

Methods: This prospective, multicenter, exploratory, pilot study aimed to investigate which parameters contribute to the occurrence of fatigue in patients with ITP. Adult patients with ITP and with or without fatigue who visited the study center for their regular appointments were asked to complete questionnaires pertaining to patient-reported outcome measures regarding bleeding symptoms, depression, sleep apnea, and hypersomnia. Blood tests included platelet count as well as different parameters like vitamin D.

Results: A total of 36 patients (100%; 27 females (75%) and nine males (25%)) with primary ITP, with a median age of 46.5 years (range 19‑83 years) were analyzed. The median duration of ITP was 4.5 years (min‑max 0-21). Approximately one-third of patients (29.4%; 10/34 patients) had no comorbidities. The two most frequently used current treatment options were "watch-and-wait" (38.9%; 14/36 patients) and "avatrombopag" (30.6%; 11/36 patients); eight patients (22.2%; 8/36 patients) needed rescue therapy with corticosteroids. There was a statistically negative correlation between fatigue and year of diagnosis (r=-0.41, p=0.014). Results indicated no statistically significant relationship between fatigue and age or differences in fatigue between the genders. Ferritin predicted fatigue with statistical significance. Platelet count was not correlated with the level of fatigue. A significant correlation was obvious between fatigue, depression, and obstructive sleep apnea syndrome (OSAS) as well as sleep-related problems (p<0.01).

Discussion: Patient characteristics were comparable to that of other studies. The level of fatigue negatively impacts the lives of patients with ITP. Age and gender were not correlated with fatigue in ITP, which is in line with other reports. Interestingly, the fatigue level was higher in patients presenting with additional depression and poor sleeping quality due to, e.g., hypersomnia, which seems common. Fatigue levels seem independent from thrombocyte levels, which were reported elsewhere.

Conclusion: Patients diagnosed with ITP several years ago cope with their condition better than patients with a more recent diagnosis, who have higher levels of fatigue. Concurrent depression, hypersomnia, and sleep apnea are important underestimated factors, which do have a negative effect on the QoL of patients with ITP. We were able to show that patients with ITP might face an unmet medical need in terms of delayed diagnosis and supportive therapy. To our knowledge, this is the first report on combined findings of depression, hypersomnia, and sleep apnea in patients with ITP.

## Introduction

Immune thrombocytopenia (ITP) is a rare disease with an incidence of two to four per 100,000 adults, fulfilling the criteria for classification of an orphan disease [1]. Low platelet counts, repeatedly measuring below 100×109/L, due to increased platelet destruction and decreased platelet production [1] lead to elevated risk of bleeding [2]. In the case of primary (idiopathic) ITP, no causative agent is identifiable [1].

Approximately 60% of adult patients with ITP experience a chronic course of the disease. In Germany, the expected annual incidence is approx. 2,400 new cases, with a prevalence of approx. 16,000 patients with chronic ITP. The number of patients potentially requiring treatment in Germany is approx. 3,500‑9,000 [1].

The treatment options for patients with ITP vary depending on the bleeding severity. They range from a "watch-and-wait approach" to corticosteroids and other treatments, e.g., intravenous immunoglobulins, immunosuppressives, thrombopoietin receptor agonists (TPO‑RAs), monoclonal antibodies, and surgical splenectomy [1].

Patients with ITP commonly exhibit symptoms such as petechiae and mucosal hemorrhages [3] and may experience cognitive symptoms [4,5], negatively affecting mental health-related quality of life (QoL) outcomes [6]. Important symptoms for patients include the effects of ITP on health-related QoL as unexplained fatigue [7], anxiety or depression, and headaches [7]. However, these symptoms may be easily overlooked [3]. The most likely multiple underlying causes of fatigue in ITP [8] have not been fully elucidated yet [2] and data on fatigue, particularly in patients with ITP, is scarce. Fatigue is a state of prolonged tiredness, exhaustion, and lack of energy that is not improved by sleep or rest [8-10]. It may be difficult to distinguish fatigue in a chronic disease setting from symptoms of depression and burnout. Improved sleeping quality, eliminating depression, increasing platelet count, and improvement of bleeding symptoms may help to alleviate fatigue [8]. Mood, belief in the disease, support from family and friends, and working ability could contribute to and be influenced by fatigue, potentially having a double-directed interplay with fatigue [2].

The QoL of patients with ITP is comparable to or even worse than that of cancer patients [1]. Hematologists may overestimate the patients’ perception of the impact of ITP on their QoL, but at the same time, they may underestimate the patients' acceptable platelet count, which can have a significant influence on treatment decisions and, thus, symptom alleviation, such as fatigue or psychological symptoms, which greatly affects the patient’ striving for a "normal" life [7].

This exploratory study aimed to investigate which parameters favor the occurrence of fatigue in patients with ITP, using several validated self-assessment questionnaires as well as laboratory parameters known to have an impact on fatigue [9-11].

## Materials and methods

The objective of this prospective, multicenter, exploratory, pilot study was to identify causes contributing to the occurrence of fatigue in patients with ITP. The study results may help to develop strategies to support patients with ITP and fatigue, aiming to improve the impact of their comorbidity. Furthermore, these findings can serve as a basis for causal research.

Patients of all genders aged ≥18 years with confirmed ITP and with or without fatigue and capable of consenting were enrolled after the provision of written informed consent. Patients presenting at the study center during their once-per-month up to once-per-quarter regular visit received their routine CBC, liver function tests, and basic metabolic panels (BMP) that include creatinine. Depending on symptoms, further parameters such as vitamin D were analyzed. There was no additional venal puncture. Parameters assessed encompassed medical history, blood count including platelet counts, CD4/CD8 ratio, vitamin D, cortisol [12], serotonin, CRP and TSH, free triiodothyronine (fT3), and free thyroxine (fT4). Due to a lack of relevance for the study, fT3 and fT4 were not included in the analysis. Therapies applied were documented. The ISTH/SSC Bleeding Assessment Score [13] was used in a modified version focusing on the patient population with ITP, i.e., leaving out hematuria, hemarthrosis, central nervous system bleeding, and other bleedings, and filled based on patients’ feedback.

In addition, patients were asked during their time in the waiting room or during the blood draw to fill out four questionnaires, focusing on physical and psychological symptoms of fatigue and the patients' QoL. The questionnaires encompassed (a) the Functional Assessment of Chronic Illness Therapy-Fatigue (FACIT‑F) questionnaire [14], a 13‑item scale assessing self‑reported fatigue and its impact upon daily activities and function; (b) the Beck Depression Inventory (BDI) [15], a self-report multiple-choice scale measuring depression based on 21 symptom domains; (c) the Epworth Sleepiness Scale [16,17], used to assess daytime sleepiness (hypersomnia; depicted as "hypersomnia" in the scale that follows), particularly in the context of clinical sleep disorder, and is a patient self-report tool rating the likelihood of falling asleep or feeling the urge to sleep during daytime in eight situations from "never" to "frequently"; and (d) the snore, tired, observed apneas, pressure, BMI, age, neck circumference, and gender (STOP-BANG) questionnaire [18], a questionnaire that asks for tiredness/fatigue in various situations (sitting, lying down, etc.). Patients answered closed yes/no questions on the covered items. Based on the STOP-BANG score, the risk for obstructive sleep apnea syndrome (OSAS) could be determined.

The measurement of parameters and provision of questionnaires were planned to be repeated every three to six months and at least twice during the study duration; actually, patients filled the questionnaires once; the measurements on CBC, liver function test, and BMP were also conducted once. Data capture was prospective. Completed questionnaires were password-protected and stored in a closed cabinet at the study center.

The study was planned to start in May 2022 and was expected to have a maximum duration of 24 months. The first patient was included in July 2022, and the last data collection was on May 25, 2023. Time since diagnosis of ITP was not restricted but was documented.

Ethics statement

The Ethics Committee at the State Medical Association of Hesse, Germany, stated formal approval, consent, and permission per the professional code of conduct for physicians on June 28, 2022 (2021-2243-evBO). This study was performed in accordance with the Declaration of Helsinki, the International Council for Harmonisation Good Clinical Practice guidelines, the General Data Protection Regulation, the German Federal Data Protection Act, and the State Data Protection Act. All participating patients provided informed consent.

Statistics

As ITP is a rare disease, participation of 35‑50 patients was estimated. We were able to recruit a small sample for this study. This is partly due to the rarity of the disease. About 60 patients are treated regularly at our center; thus, more than 50% of the patients took part in the study. It is also a pilot study where the participants were interviewed very intensively. Descriptive analysis and non-parametric statistical tests were conducted. Group comparison was carried out using the Chi-squared test, t-test, U-test, correlations, and confidence intervals. Statistical analysis was performed with R 4.3.0 (The R Foundation, Vienna, AT) [19]. A control group was not needed due to the type of hypotheses (a-h formulated: a: Fatigue in ITP is not only caused by the reduced platelet count but also by other laboratory parameters; b: Fatigue in ITP is not only caused by the reduced platelet count but also by psychological and physical concomitant circumstances, e.g., sleep apnea; c: Fatigue in ITP is not only caused by reduced platelet count but also by ITP medication (current, previous); d: Fatigue is also related to age and gender; e: The more pronounced the fatigue in the questionnaire is, the more conspicuous (pathological) the questionnaires are (similar to b); f: Fatigue in ITP is not only caused by the reduced platelet count but also by the requirement of rescue therapies; g: Comorbidities are associated with fatigue; and h: Fatigue is also influenced by disease duration).

## Results

Patient characteristics

A total of 36 patients with primary ITP were included in the final analysis set, and none of the patients were excluded from the analysis. There was no drop-out during the study. During the study period, all patients presented at the study center at least once. Four patients (11.8%) diagnosed with depression prior to this study were kept in the analysis and were well‑controlled with medication. The median age was 46.5 years (min‑max 19‑83). Twenty-seven female patients (75%) and nine male patients (25%) participated (Table 1). The median duration of ITP was 4.5 years (min‑max 0‑21). Patients could provide multiple answers regarding disease and therapy characteristics. The three most common comorbidities, based on 34 patients, were "allergy" (26.5%), "arterial hypertension," and "Hashimoto thyroiditis" (17.6% each). Nearly one-third of patients (29.4%) had no comorbidities. All patients provided information on their past and current therapies; the three most common past therapies were "corticosteroids" (47.2%), "none" (38.9%), and romiplostim (16.7%); and the most common current therapy options chosen were "watch-and-wait" (38.9%), "avatrombopag" (30.6%), "romiplostim" (19.4%), and "eltrombopag" (8.3%). Eight (22.2%) patients needed rescue therapy with corticosteroids, either prednisolone, dexamethasone, or methylprednisolone. More than one-third of patients (38.7%) did not have concurrent medication (31 of 36 patients answered this question). The three most common concurrent medications were "vitamins" (35.5%), "hormones" (25.8%), and "antihypertensive therapy" (22.6%) (Table 1).

**Table 1:** Patient characteristics a: two missing answers, b: 28 patients did not need rescue therapy, c: five missing answers, †: multiple answers were possible; therefore, numbers do not summarize to 100%, ‡: anti-diabetic treatment, FAS: full-analysis set, n: number, SD: standard deviation

	FAS
Patient characteristics	N=36
Age (years); mean (SD)	48.94 (18.91)
Gender, n (%)	
Female	27 (75)
Male	9 (25)
Duration of ITP (years); mean (SD)	5.72 (4.75)
Comorbidities, n (%)^†,a^	
None	10 (29.4)
Allergy	9 (26.5)
Arterial hypertension	6 (17.6)
Hashimoto thyroiditis	6 (17.6)
Depression	4 (11.8)
Diabetes	3 (8.8)
Dyslipidemia	3 (8.8)
Epilepsy	2 (5.9)
Rosacea	2 (5.9)
Rheumatic disorders	2 (5.9)
Past therapies, n (%)^†^	
Corticosteroids	17 (47.2)
None	14 (38.9)
Romiplostim	6 (16.7)
Eltrombopag	3 (8.3)
Fostamatinib	2 (5.6)
Immunoglobulin	2 (5.6)
Avatrombopag	1 (2.8)
Current therapy option, n (%)^†^	
Watch-and-wait	14 (38.9)
Avatrombopag	11 (30.6)
Romiplostim	7 (19.4)
Eltrombopag	3 (8.3)
Fostamatinib	3 (8.3)
Atorvastatin	1 (2.8)
Corticosteroids	1 (2.8)
Rescue therapy with corticosteroids, n (%)^†,b^	8 (22.2)
Prednisolone	4 (50.0)
Dexamethasone	2 (25.0)
Methylprednisolone	2 (25.0)
Concurrent medication, n (%)^†,c^	
None	12 (38.7)
Vitamins	11 (35.5)
Hormones	8 (25.8)
Antihypertensive therapy	7 (22.6)
Lipid-lowering drugs	4 (12.9)
Analgetic	2 (6.5)
Antiepileptic	2 (6.5)
Anti-allergic drugs	1 (3.2)
Iron	1 (3.2)
Metformin^‡^	1 (3.2)

Descriptive and correlation analysis of biomarkers

Blood samples taken during routine control were analyzed for several biomarkers (Table 2). There were no major aberrations in the laboratory values. The current median (min‑max) platelet count was 93 (2‑500)×109 L and the lowest median (min‑max) platelet count within the last year before the study was 30 (1‑141)×109 L. Descriptive statistics for biomarkers are shown in Table 2.

**Table 2:** Descriptive statistics of biomarkers a: two missing values, b: three missing values, c: four missing values, †: not in lifetime but within the last year before the study, CD4/CD8: cluster of differentiation 4/8, CRP: C-reactive protein, FAS: full-analysis set, Hb: hemoglobin, N: number, SD: standard deviation, TSH: thyroid-stimulating hormone

	FAS	
Biomarkers	N=36	Reference value
Blood count, mean (SD)		
Hb (g/dL)	13.59 (1.43)	13-18.1
Ferritin (ng/mL)	118.05 (160.88)	18-360
Platelet count (×10^9^/L)		140-336
Current	123.44 (108.24)	
Lowest^†^	42.53 (38.72)	
Vitamins, mean (SD)		
Vitamin D (ng/mL)	31.71 (18.13)	30-70
Vitamin B12 (ng/L)	365.58 (230.27)	128-648
Cortisol (µg/dL); mean (SD)^a^	13.66 (19.60)	4.3-22.4
Serotonin (ng/mL); mean (SD)^b^	55.48 (74.29)	<292
CRP (mg/L); mean (SD)	1.61 (2.15)	<3
Thyroid function tests, mean (SD)		
TSH (µg/dL)	1.24 (0.60)	0.4-4.5
CD4/CD8-ratio, mean (SD)^c^	1.78 (0.83)	<2.5

**Figure 1:**
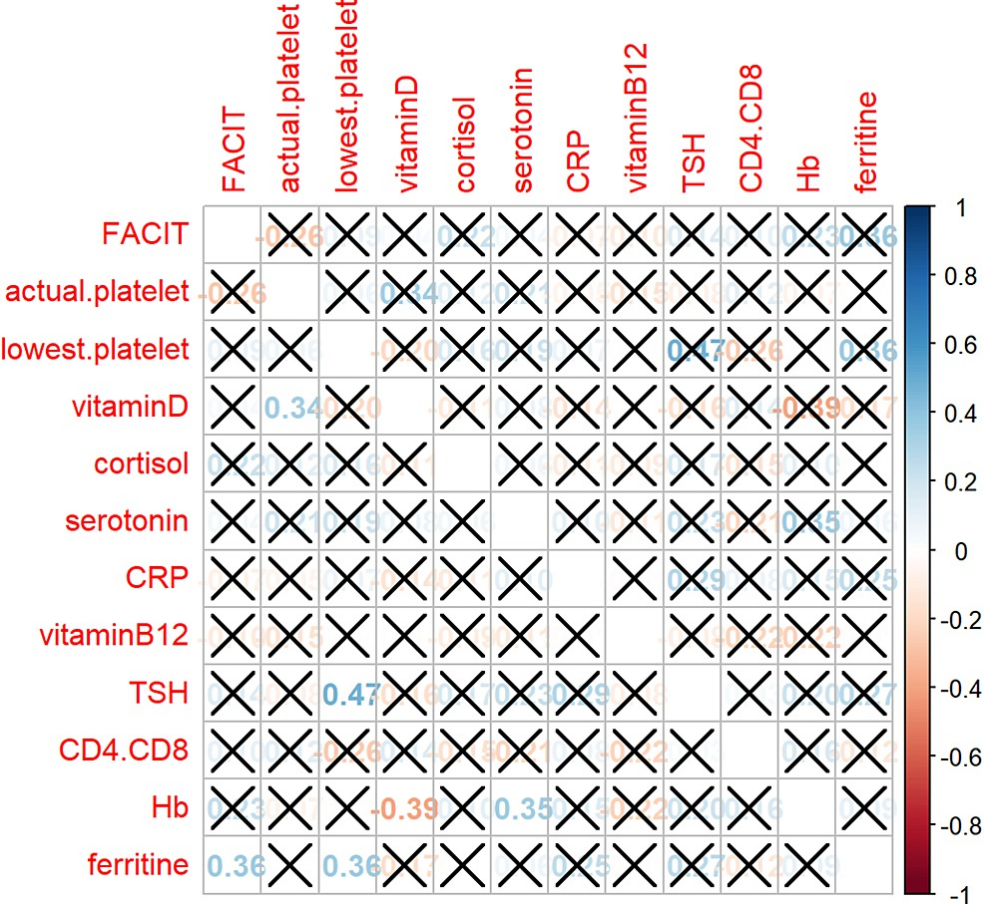
Correlation matrix between biomarkers In the upper right, above the main diagonal, adjusted p-values for multiple testing are shown, being statistically not significant. Below the diagonal, unadjusted p-values are shown. Missing values for "cortisol": two patients; for "serotonin": three patients; for CD4/CD8: four patients. CD4.CD8: a cluster of differentiation 4/8, CRP: C-reactive protein, FACIT‑F: Functional Assessment of Chronic Illness Therapy–Fatigue, Hb: hemoglobin, TSH: thyroid-stimulating hormone

A correlation analysis was performed to investigate the relationship between fatigue and biological markers (Table 3, Figure 1).

**Table 3:** Correlation analysis of FACIT-F and biomarkers (FAS) *p<0.05; **p<0.01; values in parentheses indicate the 95% CI for each correlation. †: not in a lifetime but within the last year before the study, a: two missing values, b: three missing values, c: four missing values, CD4/CD8: cluster of differentiation 4/8, CI: confidence interval, CRP: C-reactive protein, FAS: full-analysis set, Hb: hemoglobin, N: number, Plt #: platelet count, TSH: thyroid-stimulating hormone

Variable	FACIT-F	Current plt #	Lowest plt #^†^	Vitamin D	Corti-sol^a^	Sero-tonin^b^	CRP	Vitamin B12	TSH	CD4/ CD8^c^	Hb	Ferritin
						Correlation r (95% CI)						
FACIT-F												
Current plt #	-0.26											
	(-0.54, 0.07)											
Lowest plt #^†^	0.09	0.06										
	(-0.25, 0.40)	(-0.27, 0.38)										
Vitamin D	0.04	0.34*	-0.2									
	(-0.29, 0.36)	(0.01, 0.60)	(-0.49, 0.14)									
Cortisol^a^	0.22	0.12	0.16	-0.11								
	(-0.13, 0.52)	(-0.23, 0.44)	(-0.19, 0.47)	(-0.44, 0.23)								
Serotonin^b^	0.04	0.21	0.19	0.08	0.06							
	(-0.31, 0.38)	(-0.15, 0.51)	(-0.16, 0.50)	(-0.27, 0.41)	(-0.29, 0.39)							
CRP	-0.07	-0.05	0.07	-0.14	-0.11	0.1						
	(-0.39, 0.26)	(-0.38, 0.28)	(-0.26, 0.39)	(-0.45, 0.19)	(-0.43, 0.24)	(-0.25, 0.43)						
Vitamin B12	-0.1	-0.15	0	-0.01	-0.09	-0.11	-0.01					
	(-0.42, 0.23)	(-0.45, 0.19)	(-0.32, 0.33)	(-0.33, 0.32)	(-0.42, 0.25)	(-0.44, 0.24)	(-0.34, 0.32)					
TSH	0.14	-0.08	0.47**	-0.16	0.17	0.23	0.29	-0.08				
	(-0.20, 0.45)	(-0.40, 0.26)	(0.17, 0.69)	(-0.47, 0.18)	(-0.18, 0.48)	(-0.13, 0.53)	(-0.05, 0.56)	(-0.40, 0.25)				
CD4/CD8^c^	0.1	0.12	-0.26	0.14	-0.15	-0.21	0.08	-0.22	0.03			
	(-0.26, .043)	(-0.24, 0.45)	(-0.55, 0.10)	(-0.22, 0.46)	(-0.49, 0.22)	(-0.53, 0.16)	(-0.28, 0.41)	(-0.53, 0.14)	(-0.32, 0.38)			
Hb	0.23	-0.07	0	-0.39*	0.1	0.35*	0.15	-0.22	0.2	0.16		
	(-0.10, 0.52)	(-0.39, 0.27)	(-0.33, 0.33)	(-0.64, -0.08)	(-0.24, 0.43)	(0.00, 0.62)	(-0.19, 0.46)	(-0.51, 0.11)	(-0.13, 0.50)	(-0.20, 0.48)		
Ferritin	0.36*	-0.01	0.36*	-0.17	-0.01	0.06	0.25	-0.01	0.27	-0.12	0.09	
	(0.03, 0.61)	(-0.34, 0.32)	(0.03, 0.61)	(-0.47, 0.17)	(-0.34, 0.33)	(-0.29, 0.40)	(-0.09, 0.53)	(-0.34, 0.32)	(-0.07, 0.55)	(-0.45, 0.24)	(-0.25, 0.41)	

Adjusted p-values showed no statistical significance. With unadjusted p-values, ferritin was the only biomarker with a statistically significant correlation with the FACIT‑F score (r=0.36, p=0.032). Higher ferritin levels led to higher FACIT‑F scores and, thus, less fatigue. Interestingly, the platelet count was not correlated with the level of fatigue.

Analysis of patient questionnaires

The response rate for the questionnaires was high with all 36 patients filling the forms.

Reliability of questionnaires

The internal consistency of the questionnaires’ scores and scales are shown in Table 4. Cronbach’s alpha was lowest for the STOP-BANG questionnaire (α=0.55, not reliable) and highest for the FACIT-F questionnaire (α=0.95, very reliable). The BDI, hypersomnia, and OSAS questionnaires showed very good or good reliability, respectively. Most items of the ISTH/SSC Bleeding Assessment Score appeared to be constant values, i.e., due to practically no variance; reliability was questionable (α=0.063). Due to the high uncertainty detected, both the STOP-BANG questionnaire and the ISTH/SSC Bleeding Assessment Score are not reported further because drawing meaningful statistical inference implications is impossible.

**Table 4:** Internal consistency of the total score, trial outcome index, and subscales (FAS) †: reliability of the ISTH/SSC Bleeding Assessment Score could not actually be calculated as the items had no variance. BDI: Beck Depression Inventory, FACIT‑F: Functional Assessment of Chronic Illness Therapy–Fatigue, FAS: full-analysis set, ISTH/SSC: International Society on Thrombosis and Haemostasis/Scientific and Standardization Committee, n: number, OSAS: obstructive sleeping apnea syndrome, SD: standard deviation, STOP-BANG: snore, tired, observed apneas, pressure, BMI, age, neck circumference, and gender

Scale	Items (n)	Range of scores	Mean (SD)	Alpha
FACIT‑F	13	4-47	1.67 (0.99)	0.95
BDI	21	0-26	0.54 (0.51)	0.93
Hypersomnia	8	3-19	0.85 (0.45)	0.83
OSAS	8	2-22	1.09 (0.62)	0.83
STOP-BANG	5	1-6	0.26 (0.20)	0.55
ISTH/SSC Bleeding Assessment Score	10	0-56	0.17 (0.10)^†^	0.063^†^

Descriptive results of FACIT-F, BDI, hypersomnia, and OSAS

The FACIT‑F questionnaire used to collect information on the presence/absence of fatigue showed very high internal consistency reliability (Tables 4-5). Patients could rate the 13 items on fatigue from "not at all" to "very high," the response applying to their status past seven days. The higher the score, the better the patients' QoL is, while values below 30.0 clearly indicate the presence of fatigue. This was observed for more than one-third of patients (13 patients; 36.1%). The mean FACIT‑F score with SD was 34.00 (12.91) (Table 5).

**Table 5:** Questionnaires on psychologic and bodily symptoms – correlation analysis (FAS) **p<0.01; BDI: Beck Depression Inventory, CI: confidence interval, FACIT‑F: Functional Assessment of Chronic Illness Therapy–Fatigue, FAS: full-analysis set, n: number, OSAS: obstructive sleeping apnea syndrome, SD: standard deviation

		FACIT-F	Hypersomnia	OSAS
Variable	Mean (SD)	(95% CI)	(95% CI)	(95% CI)
FACIT-F	34.00 (12.91)			
Hypersomnia	11.33 (5.54)	-0.79**		
		(-0.89, -0.62)		
OSAS	8.69 (4.96)	-0.55**	0.52**	
		(-0.75, -0.28)	(0.23, 0.72)	
BDI	11.36 (10.90)	-0.79**	0.70**	0.26
		(-0.89, -0.63)	(0.48, 0.84)	(-0.08, 0.54)

Table 5 shows that FACIT-F has a strong negative correlation with hypersomnia and BDI. This means that higher fatigue scores are associated with higher sleepiness and depression scores. The correlation coefficients are -0.79 and -0.79, respectively, and both are significant at the 0.01 level.

Hypersomnia has a moderate positive correlation with OSAS and BDI. This means that higher sleepiness scores are associated with higher apnea and depression scores. The correlation coefficients are 0.52 and 0.70, respectively, and both are significant at the 0.01 level.

OSAS has a weak positive correlation with BDI. This means that higher apnea scores are slightly associated with higher depression scores. The correlation coefficient is 0.26, but it is not significant at the 0.01 level.

These results suggest that fatigue, sleepiness, apnea, and depression are interrelated variables that affect each other in different ways.

The BDI questionnaire, which allows the collection of symptoms of mental disorders/depression, based on 21 symptom domains, had also very good internal consistency (Table 2). Information on symptom domains is collected per groups of statements, from which the most applicable is to be selected. BDI scores can range from 0 to 26, and scores <8 are considered unremarkable, scores from 14 to 19 are considered a mild to moderate expression of depressive symptoms, and scores ≥20 are considered clinically relevant. Eighteen patients (50.0%) had no depression, nearly one-fourth of patients (22.2%) had minimal depression, and 11.1% of patients (each four) experienced a mild or moderate form of depression. Two patients had a severe depression.

We collected information on hypersomnia using the Epworth Sleepiness Scale (hypersomnia) and sleeping apnea using the OSAS questionnaire, both showing good reliability (Table 4). The hypersomnia questionnaire retrospectively captures subjective rating of the probability of (actually) falling asleep in eight given typical situations, using a scale from 0 (unlikely) to 3 (very likely); the total score may range from 9 to 19. The height of the score is associated with increased sleepiness. Hypersomnia was quite likely in 11 patients (30.6%); we observed no aberrance in the other patients.

Scores derived from the OSAS questionnaire depict the OSAS risk. More than one-fourth of patients (11 patients; 30.5%) had an elevated risk for OSAS or presented with "suspicion of mild to moderate sleep-related breathing disorder." Two patients (5.6%) had a severe sleep disorder with health risks.

Relationship between fatigue, year of diagnosis, age, and gender using correlation analysis

Results indicated a statistically negative correlation between the year of diagnosis and FACIT‑F score (r=-0.41, p=0.014). Patients, who had been diagnosed with ITP several years ago had higher FACIT‑F scores, which indicate a lower level of fatigue. Thus, patients with a very recent diagnosis presented with more severe levels of fatigue. The analysis of a potential relationship between fatigue and age indicated no statistically significant relationship (r=0.14, p=0.4189). To explore potential differences in the manifestation of fatigue between genders, a Mann‑Whitney U test, as a non-parametric test was used, due to the small and relatively diverse sample size. There was no statistically significant difference in fatigue between the genders (U=161.50, p=0.149). A descriptive analysis is shown in Table 6.

**Table 6:** Descriptive statistics of the relationship between FACIT-F and gender (FAS) FAS: full-analysis set, min‑max: minimum‑maximum, n: number, SD: standard deviation

	Females	Males
Scale	(n=27)	(n=9)
Mean (SD)	32.30 (13.31)	39.11 (10.69)
Median (min-max)	36 (4-50)	42 (20-50)

Correlation analysis of FACIT-F and other questionnaires

**Figure 2:**
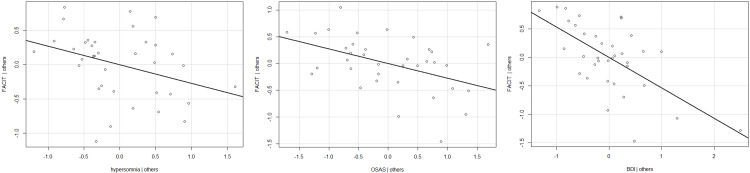
Added variable plots for the questionnaires on hypersomnia, OSAS, and BDI The regression line reflects the standardized regression coefficient, i.e., the correlation of hypersomnia, OSAS, or BDI, respectively, with the FACIT-F if controlled for the respective other selectors. BDI: Beck Depression Inventory, FACIT-F: Functional Assessment of Chronic Illness Therapy–Fatigue, OSAS: obstructive sleeping apnea syndrome

The analysis of statistical inference of the FACIT-F, hypersomnia, OSAS, and BDI questionnaires is shown in Table 7. The Shapiro-Wilk test showed no deviation from the normal distribution (p=0.655). There were no outliers because Cook distances with a maximum of 0.32 were all <1 and the Breusch-Pagan test did not indicate heteroskedasticity (p=0.092). Since several predictors were included in the regression model, multicollinearity between the predictors had to be tested using variance inflation factors (vif). Multicollinearity was not a problem because the vifs with a maximum of 2.57 were all <10. In the regression analysis, the relative influence of variables is taken into account, i.e., beyond the other variables, all individual predictors assert significant variance in the FACIT‑F questionnaire. All three predictors were able to significantly predict fatigue with the FACIT‑F score (Table 5): The stronger the hypersomnia symptoms, the lower the FACIT‑F score and thus the higher the level of fatigue; the pattern was observed for OSAS and BDI scores. The multiple linear regression is shown in Table 7. Added variable plots on the predictors are shown in Figure 2.

**Table 7:** Multiple linear regression analysis for the prediction of FACIT-F (FAS) BDI: Beck Depression Inventory, FACIT‑F: Functional Assessment of Chronic Illness Therapy–Fatigue, FAS: full-analysis set, OSAS: obstructive sleeping apnea syndrome, SD: standard deviation

Scale	Estimate β (SD)	t-value	p-value
Hypersomnia	-0.27 (0.13)	-2.10	0.043
OSAS	-0.28 (0.96)	-2.87	0.007
BDI	-0.53 (0.11)	-4.65	<0.001

Interestingly, coping seems to play an important role in patients diagnosed with ITP for a longer time frame. Patients more recently diagnosed experience higher levels of fatigue more often, which may indicate that patients need many months and even years to adapt to their special situation. More than one-third of patients had fatigue. Interestingly, half of our patients presented with signs of depression ranging from minimal to severe. This may indicate that the number of patients with ITP and concurrent depression, with its negative effects on QoL, may be underestimated. We could show a statistically significant correlation between fatigue and depression. Surprisingly, OSAS in various forms, from borderline values to severe sleep disorder with health risk, was observed in most patients (66.7%), and more than one-third of patients were likely to have hypersomnia. To our knowledge, this is the first study dealing with this topic. This aspect has not yet been studied in detail in patients with ITP. A connection to fatigue is postulated.

ITP poses a burden on the patients' lives: medical and psychological support by diagnosis may be perceived as insufficient. Patients with ITP endure both emotional and clinical burdens as a result of their condition. Follow-up studies are needed to strengthen the knowledge of the clinical importance of fatigue, depression, and hypersomnia/OSAS in patients with ITP.

Correlation analysis of FACIT-F and current, previous, and rescue medication

Several patients took several current medications. Due to diversity and low patient numbers in the single medication subgroups, a Kruskal-Wallis test was performed to investigate differences in fatigue between different current ITP medications (Table 1). There were no statistically significant differences between the single "current medication" groups (χ²=3.81, p=0.702). However, no meaningful deduction was possible due to small sample sizes, such as having only one patient per group. Group comparison of the two groups with >10 patients, "watch‑and‑wait" (n=14) and "avatrombopag" (n=11), showed no statistically significant differences (Mann-Whitney U test: U=85.5; p=0.661). Yet, patients in the "avatrombopag" group partly took further current medication, which may influence results, as the isolated effects of both medications cannot be compared. The same setting, high diversity, and low sample sizes were applied for the analyses of previous (χ²=3.55; p=0.939) and rescue medication (χ²=0.13, p=0.939); thus, there were no statistically significant differences between groups regarding a potential correlation with FACIT‑F score.

Correlation analysis of FACIT-F and comorbidities and comedication

Patients showed a high diversity in comorbidities (Table 1), leading to a wide range of subgroups with low patient numbers. Results were similar when compared to the individual medication groups. There were no statistically significant differences between the individual "comorbidity" groups (χ²=22.31, p=0.173). The same applied to the "comedication" groups (Table 1; χ²=20.8, p=0.235) regarding a potential effect on FACIT‑F scores. Comparison of the largest groups "none" and "vitamins" showed no statistically significant differences (U=83; p=0.308).

Correlation analysis of biomarkers and questionnaires

We investigated even the correlation between biomarkers and all questionnaires, except for the FACIT-F questionnaire (Table 5; see correlation including FACIT-F questionnaire: Table 3) as part of an explorative analysis. There was a positive statistically significant correlation between hemoglobin (p=0.017) and vitamin D and between hypersomnia and ferritin (p=0.036). Vitamin D level (p=0.042) and current platelet count, BDI (p=0.008) and current platelet count, ferritin (p=0.032) and lowest platelet count within the last year before the study, TSH (p=0.003) and lowest platelet count within the last year before the study, hemoglobin (p=0.049), and serotonin were statistically significantly negatively correlated. However, none of the parameters with internal correlation was associated with the level of fatigue but ferritin (Table 3).

## Discussion

Our findings on demographic characteristics of patients with ITP, i.e., age, gender, and mean duration of ITP were consistent with findings by other publications [3,7,8]. In middle age, women are more likely to develop ITP than men [1]. Patients with ITP may have a higher prevalence of comorbid diseases, such as diabetes prior to diagnosis of ITP, compared to people without ITP [20]. Older patients (>60 years) with ITP often present with, for example, hypertension, diabetes, coronary artery disease, neuropsychiatric diseases, and others. Patients with ITP have an increased risk of developing malignant disease compared to people without ITP [1]. It seems that the age of ITP onset increased in the past few years to approx. 60 years [1]. Patients with ITP may also experience cognitive impairment, which is interestingly unrelated to platelet count but obviously negatively impacting QoL [4,5]. Approximately one-third of the patients in our study had no comorbidities, which may be due to the relatively low median age of 46 years. The most common treatments in our patient population partly differed from the ITP-World Impact Survey (I-WISh), an exploratory, cross-sectional survey on the multiform effects of ITP and its treatments on patients' lives [3]. In our study, most patients adopted a "watch-and-wait" approach (39%; 14/36 patients), followed by two TPO-RAs (together 50%; 18/36 patients), the former being rather prescribed by physicians during the first half year following diagnosis [3]. For patients with persistent, chronic, or recurrent ITP, TPO-RAs [1] are frequently prescribed [3]. The "watch-and-wait" approach may be chosen in case of minor bleeding [1], which is consistent with our patient population.

The common and distressing symptom of ITP, fatigue, remains consistently severe over time and, independent of the patients' age, does not appear to correlate to platelet counts [9]. This means that with an increased platelet count, even after TPO-RA application [2,9], fatigue may not necessarily be alleviated [4]. In our correlation analysis, we did not find any statistically significant correlation of fatigue with either the current or lowest platelet count within the last year before the study, which is in contrast to findings from other studies [8,21]. However, understanding the influence of platelet count is not entirely clear [2], and results from different studies may be contradictory [20]. In our study, only ferritin showed a statistically significant association with the FACIT‑F score (p=0.032). Low ferritin levels were associated with severe fatigue in adolescents with heavy menstrual bleeding, as described before [10].

Fatigue, commonly diagnosed in chronic diseases [2], is a frequent and severe comorbidity observed in patients with ITP [3,4,7,11]. It negatively affects not only bodily functioning but also mental health [2,7] and leads to problems in coping with the challenges of daily living and decreases health-related QoL [3,22]. Our correlation analysis on FACIT‑F scores and time point of diagnosis revealed that a more recent diagnosis was statistically significantly related to more severe fatigue symptoms. Our mean FACIT‑F score was comparable to the one reported by another publication [8]. FACIT‑F score seems to be uncorrelated with age [8,9,21] and gender [9,21], which conforms to our findings. A monocenter study that included adults with ITP and a considerably lower median (min‑max) platelet count (35 (1‑350)×109/L) than reported by us (93 (2‑500)×109 L) detected a statistically significant correlation of higher fatigue levels in patients with more severe bleeding and low platelet counts. A significant correlation between depressive mood and anxiety, measured by the Hospital Anxiety and Depression Scale (HADS-D/A), and poor quality of sleep, measured by the Pittsburgh Sleep Quality Index (PSQI), encompassing subjective sleep quality, sleep latency, sleep duration, habitual sleep efficiency, sleep disturbances, use of sleeping medication, and daytime dysfunction, and fatigue level was described [8]. These findings, using different questionnaires with partly different items addressed, point in the direction of our study. Fatigue as well as decreased energy levels and activity restrictions can be frustrating for patients. Impaired sleep and mood can be associated with fatigue in chronic disease. Daytime sleepiness, also observed here, has been previously reported in a multivariate model to be independently associated with fatigue in patients with ITP without bleeding, in addition to low platelet counts below 30.000/µL, bleeding, presence of other diagnosed medical conditions, and orthostatic symptoms [21].

Most patients with ITP in the I-WISh cross-sectional survey, which included 1,507 patients and 472 physicians, experienced reduced energy levels (85%), decreased capacity to exercise (77%), and a negative impact of ITP on their ability to perform daily tasks (75%). Half of them felt a substantial impact on emotional well-being (49%), and 63% were afraid of worsening disease conditions or premature death. Employment was negatively affected, with some patients feeling the need to reduce working hours or terminate employment, along with reduced work productivity. Similar impacts were observed for daily activities, e.g., childcare, exercise, or studying. Noteworthy, patients' physicians acknowledged that health-related QoL in patients with ITP was substantially reduced [22]. Patients rated fatigue among the most frequent, severe symptoms associated with ITP at diagnosis (58% most frequent; 73% most severe), although physicians assigned it a lower priority (30%). Fatigue was the top symptom patients wanted resolved (46%) and still persisted at the completion of the I‑WISh survey [3]. The I-WISh survey did not use a depression scale.

The BDI questionnaire, recording symptoms of mental disorders/depression, showed that a surprisingly high proportion of patients, half of patients (18/36 patients), answered that they had symptoms of depression, varying from minimal to severe. Prior to this study, only four patients (11.1%) had shown signs of depression. This finding is important as it may identify an unmet need in this patient population, as adequate treatment for addressing depression may be delayed or not received at all. Previous studies mostly focused on QoL corresponding questionnaires [3,6,9,22,23] and rarely used a depression questionnaire. Another study also showed that depression or anxiety was reported by patients with ITP [20]. Patients with chronic diseases, such as ITP or cancer, may experience depression, sleep disturbance, and fatigue at the same time in addition to underlying diseases [20]. Our data can confirm this hypothesis. Daytime sleepiness was found to correlate with fatigue in univariate analysis in another recent survey [21]. Sleep deprivation and daytime sleepiness have a negative impact on perceived QoL and may also impact signs of depression and anxiety in individuals affected [24]. OSAS may be bidirectionally related to comorbidities, e.g., heart failure, metabolic syndrome, and stroke [25], highlighting the importance of pursuing our findings in patients with ITP.

To the best of our knowledge, this is the first study to address the finding that patients with ITP have symptoms of sleeping disorders such as OSAS and hypersomnia, as well as fatigue and signs of depression.

Limitations

Any self-reported results may be influenced by the health status of the patients on the survey day. The motivation or way of completing questionnaires can differ between patients. Most data collected with the questionnaires were patient-reported data and, therefore, not validated by a physician. Due to logistical reasons, questionnaires were handed out to patients on one occasion only. Because ITP is a rare disease, the number of patients included in our study was low, although we have reached our (lower) recruitment target of 35 patients. Due to the small number of patients and the fact that some of the patients took several medications, a statistically reliable statement as to whether there was a correlation between fatigue and a therapy strategy was impossible, which suggests that this trial has no control arm; some of the findings may be found in a non-ITP population (e.g., relationship with sleep apnea).

During the COVID‑19 pandemic, the importance of risk factor laboratory values, such as tumor necrosis factor-α and interleukin-6 levels, has grown, but these were not planned to be assessed in our study. Due to the COVID‑19 pandemic, it was not possible to examine sleeping apnea by screening in a sleeping laboratory. Therefore, we have chosen the Epworth Sleepiness Scale to assess hypersomnia and the STOP-BANG and OSAS questionnaires to assess sleep apnea.

## Conclusions

Interestingly, coping seems to play an important role in patients diagnosed with ITP for a longer time frame. Patients more recently diagnosed experience higher levels of fatigue more often, which may indicate that patients need many months and even years to adapt to their special situation. More than one‑third of patients had fatigue. Interestingly, half of our patients presented with signs of depression ranging from minimal to severe. This may indicate that the number of patients with ITP and concurrent depression, with its negative effects on QoL, may be underestimated. We could show a statistically significant correlation between fatigue and depression. Surprisingly, OSAS in various forms, from borderline values to severe sleep disorder with health risk, was observed in most patients (66.7%), and more than one-third of patients were likely to have hypersomnia. To our knowledge, this is the first study that dealt with this topic. This aspect has not yet been studied in detail in patients with ITP. A connection to fatigue is postulated.

ITP poses a burden on patients’ lives: medical and psychological support by diagnosis may be perceived as insufficient. Patients with ITP endure both emotional and clinical burdens as a result of their condition. Follow-up studies are needed to strengthen the knowledge of the clinical importance of fatigue, depression, and hypersomnia/OSAS in patients with ITP.
